# Milk microbiome transplantation: recolonizing donor milk with mother's own milk microbiota

**DOI:** 10.1007/s00253-023-12965-8

**Published:** 2024-01-09

**Authors:** Lisa F. Stinson, Jie Ma, Ching Tat Lai, Alethea Rea, Sharon L. Perrella, Donna T. Geddes

**Affiliations:** 1 School of Molecular Sciences, The University of Western Australia, Perth, Australia; 2 Mathematics and Statistics, Murdoch University, Perth, Australia

**Keywords:** Human milk, Microbiome, Holder pasteurization, UV-C, Donor milk, Recolonization

## Abstract

**Abstract:**

Donor human milk (DHM) provides myriad nutritional and immunological benefits for preterm and low birthweight infants. However, pasteurization leaves DHM devoid of potentially beneficial milk microbiota. In the present study, we performed milk microbiome transplantation from freshly collected mother’s own milk (MOM) into pasteurized DHM. Small volumes of MOM (5%, 10%, or 30% v/v) were inoculated into pasteurized DHM and incubated at 37 °C for up to 8 h. Further, we compared microbiome recolonization in UV-C-treated and Holder-pasteurized DHM, as UV-C treatment has been shown to conserve important biochemical components of DHM that are lost during Holder pasteurization. Bacterial culture and viability-coupled metataxonomic sequencing were employed to assess the effectiveness of milk microbiome transplantation. Growth of transplanted MOM bacteria occurred rapidly in recolonized DHM samples; however, a greater level of growth was observed in Holder-pasteurized DHM compared to UV-C-treated DHM, potentially due to the conserved antimicrobial properties in UV-C-treated DHM. Viability-coupled metataxonomic analysis demonstrated similarity between recolonized DHM samples and fresh MOM samples, suggesting that the milk microbiome can be successfully transplanted into pasteurized DHM. These results highlight the potential of MOM microbiota transplantation to restore the microbial composition of UV-C-treated and Holder-pasteurized DHM and enhance the nutritional and immunological benefits of DHM for preterm and vulnerable infants.

**Key points:**

*• Mother’s own milk microbiome can be successfully transplanted into donor human milk.*

*• Recolonization is equally successful in UV-C-treated and Holder-pasteurized milk.*

*• Recolonization time should be restricted due to rapid bacterial growth.*

**Graphical Abstract:**

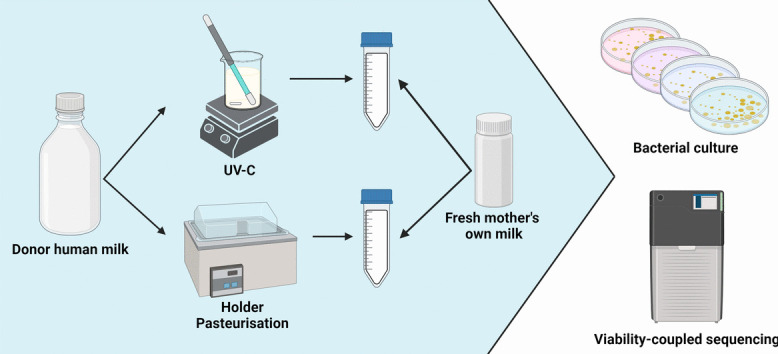

**Supplementary Information:**

The online version contains supplementary material available at 10.1007/s00253-023-12965-8.

## Introduction

Consumption of human milk is a strong determinant of the composition of the early-life microbiome (Stewart et al. 2018), with formula-fed infants harboring a gut microbial community distinctly different to their breastfed counterparts (Ma et al. 2020; Parnanen et al. 2022; Wang et al. 2020). Where mother’s own milk (MOM) is not available, donor human milk (DHM) may be given as an alternative. This practice is particularly common among mothers of preterm and low birthweight (LBW) infants, who may be unable to produce sufficient volumes of milk to meet their infants’ needs (Tran et al. 2020). MOM is the gold standard of nutrition for this at-risk group of infants, with MOM-fed infants experiencing lower risk of sepsis and necrotizing enterocolitis (NEC) and shorter hospital stays (Altobelli et al. 2020; Dritsakou et al. 2016a; Dritsakou et al. 2016b; Mannel and Peck 2018; Miller et al. 2018; Schanler et al. 1999). Where MOM volumes are insufficient to meet an infant’s needs, DHM confers significant health benefits compared to infant formula, including reduced risk of necrotizing enterocolitis (NEC) (Quigley et al. 2018). However, to ensure food safety, DHM is routinely pasteurized in milk banks, leaving it devoid of potentially important microbiota. Given evidence that maternal microbes may be vertically inherited via human milk (Asnicar et al. 2017; Duranti et al. 2017; Jost et al. 2014; Milani et al. 2015) and that MOM bacteria contribute to the nutritional benefits of milk via production of proteases and other metabolic enzymes (Dallas et al. 2015), pasteurization of DHM may alter infant microbiome colonization patterns and infant health. Indeed, the gut microbiome of DHM-fed infants differs significantly from those fed MOM, with reduced levels of Bifidobacteria and increased levels of *Staphylococcus* and *Pasteurellaceae* consistently observed (Parra-Llorca et al. 2018; Pineiro-Ramos et al. 2021). These differences may contribute to the different health outcomes observed in DHM-fed infants compared to MOM-fed infants (Cartagena et al. 2022; Hard et al. 2019) and may have consequences for establishment of the early-life microbiome.

Given the absence of maternal microbiota in pasteurized DHM, recent studies have attempted to recolonize DHM with MOM microbes. In these studies, small volumes of MOM (ranging from 1 to 30% v/v) were inoculated into Holder-pasteurized DHM (Cacho et al. 2017; Mallardi et al. 2021; Torrez Lamberti et al. 2021). The bacterial culture results of these proof-of-concept studies have demonstrated that MOM bacteria are able to expand in Holder-pasteurized DHM over 4–8 h of incubation. However, the culture-independent arms of these studies were confounded by the presence of DNA from pasteurized bacteria in DHM samples. As metataxonomic techniques such as 16S rRNA gene sequencing are unable to differentiate between DNA from viable and non-viable organisms, this type of analysis is obscured by endogenous DNA in DHM samples. Indeed, in all three studies, the level of alpha diversity was the same in the pasteurized DHM and MOM samples. DNA-based metataxonomic methods such as 16S rRNA gene sequencing are not able to differentiate between DNA from viable and non-viable bacteria (Emerson et al. 2017). These studies were therefore unable to demonstrate that the MOM microbial profile could be restored in pasteurized DHM. We have recently pioneered the use of propidium monoazide (PMA; a cell-membrane-impermeable DNA-intercalating dye) to assess the viability of bacteria in fresh (Stinson et al. 2021) and cold-stored (Stinson et al. 2022) human milk. Our work demonstrated that many of the bacterial sequences in human milk derive from non-viable cells. This has important implications when assessing the success of recolonization attempts using metataxonomic techniques.

In the present study, we have utilized bacterial culture and PMA-coupled full-length 16S rRNA gene sequencing to evaluate the ability of viable MOM microbes to recolonize pasteurized DHM. In addition to assessing Holder-pasteurized DHM, we have also assessed UV-C-treated DHM. Recent studies have demonstrated that UV-C treatment is able to reduce bacterial titers in DHM in a similar fashion to Holder pasteurization, while also conserving many of the important bioactive components of milk (Christen et al. 2013a; Christen et al. 2013b). This makes UV-C-treated milk a promising target for recolonization, as it more closely resembles the biochemical composition of unpasteurized milk. Further, preservation of bioactives such as antimicrobial peptides, immunoglobulins, and lipases, may confer health benefits to preterm and LBW infants. Recolonized UV-C-treated DHM may thereby best imitate the biochemical and microbiological composition of MOM.

## Materials and methods

### Study design

**Fig. 1:**
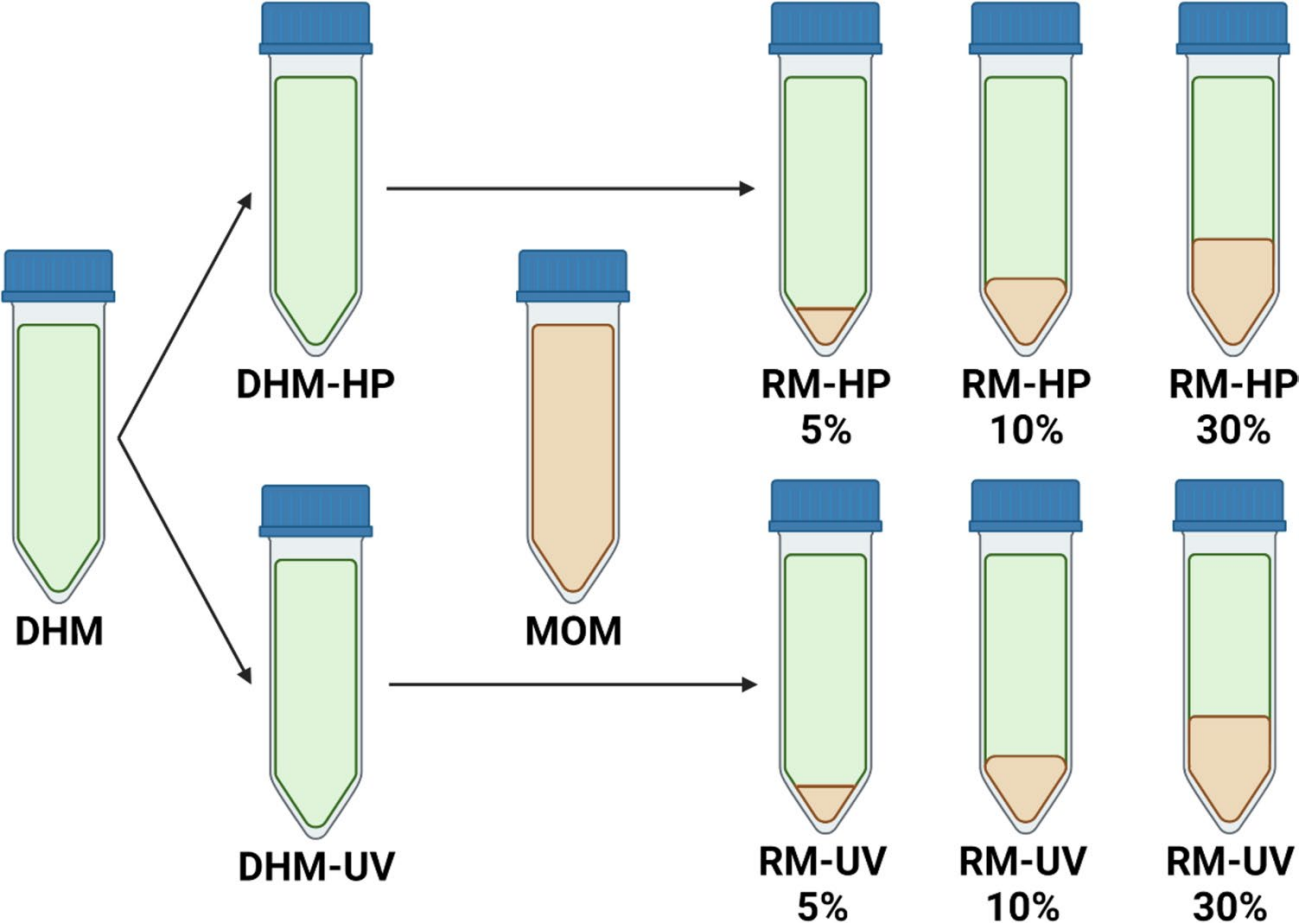
Study design. Donor human milk (DHM) samples were split into two equal aliquots: one for Holder pasteurization (DHM-HP) and one for UV-C treatment (DHM-UV). Treated samples were then inoculated with freshly collected mother’s own milk (MOM) at volumes of 5%, 10%, and 30% to create recolonized milk (RM)

Donor human milk (DHM) samples (*n*=3) were collected and split into two equal aliquots: one for Holder pasteurization (DHM-HP) and one for UV-C treatment (DHM-UV). Mother’s own milk samples (*n*=9) were freshly collected and inoculated into DHM-HP and DHM-UV samples at 5%, 10%, and 30% v/v ratios (Fig. 1). Recolonized milk (RM) samples were incubated at 37 °C for 8 h, with samples taken at 0, 4, and 8 h. MOM samples and un-inoculated DHM-HP/UV samples were incubated alongside the RM samples as positive and negative controls, respectively. Each batch of DHM was recolonized with three different MOM samples, giving a total of nine recolonization experiments. All participants gave informed written consent, and the study was approved by the University of Western Australia (RA4204023).

### Collection and pasteurization of donor milk samples

Mothers with large volumes of frozen stored breast milk were invited to donate milk to this study. We required a total of 800 mL per batch (*n*=3 batches) of donor milk to allow 400 mL for each pasteurization technique. Given these large volumes, it was necessary to combine milk from two donors in order to achieve the target volume for one batch. However, as combining milk from different donors is routinely performed in donor milk banks, this approach was reflective of clinical practice. The other two batches consisted of milk from single donors.

Each batch of DHM was divided into two equal aliquots of 400 mL. One aliquot was Holder-pasteurized by heating in a water bath to 62.5 °C for 30 min. Temperature was monitored using a thermal probe placed within a bottle of human milk. The other aliquot was UV-C-treated as previously described (Stinson et al. 2023). After treatment, 1-mL samples were taken for bacterial culture, and the remaining volume was divided into 75-mL aliquots and frozen at −80 °C.

### Collection of mother’s own milk (MOM) samples

Mothers (*n*=9) attended a study visit at our facilities to express 30 mL of milk (MOM) using their own breast pumps. Samples were used immediately (<10 min) to recolonize DHM-HP and DHM-UV samples.

### Recolonization of donor milk with MOM

DHM-HP and DHM-UV samples were thawed at 4 °C then inoculated with MOM at ratios of 5%, 10%, and 30% v/v (RM-HP 5%, RM-HP 10%, RM-HP 30%, RM-UV 5%, RM-UV 10%, RM-UV 30%). MOM, DHM-HP, DHM-UV, and all RM samples were incubated at 37 °C for 8 h. Samples were taken for bacterial culture and sequencing at 0, 4, and 8 h.

### Bacterial culture

Quantification of bacterial titers was performed by plating out serial dilutions of each sample onto various culture media. Culture media and conditions were selected based on the most common groups of bacteria cultivated from human milk: acidified de Man, Rogosa, and Sharpe (MRS) agar for lactic acid bacteria, Wilkins Chalgren for anaerobic bacteria, Mannitol salt agar (MSA) for Staphylococcus, and nutrient agar for facultative aerobes. MRS and Wilkins Chalgren agar plates were incubated anaerobically for 48 h, and MSA and nutrient agar plates were incubated aerobically for 48 h.

### Viability-coupled 16S rRNA gene sequencing

For each sample at each time point, a 1-mL aliquot was taken for viability-coupled 16S rRNA gene sequencing. Each aliquot was centrifuged at 10,000 × *g* for 10 min at 4 °C, and the fat and supernatant were discarded. Pre-treatment with PMA was performed prior to DNA extraction as previously described (Stinson et al. 2021). PMA is a cell-membrane-impermeable DNA-intercalating dye that prevents amplification and sequencing of DNA that derives from non-viable cells. Bacterial DNA profiles generated from PMA-treated aliquots thereby represent the viable microbiota only.

DNA was extracted using the QIAGEN MagAttract Microbial DNA kit on the King Fisher Duo. The full-length bacterial 16S rRNA gene was amplified using UNI-tagged 27F/1492R primes as previously described (Stinson et al. 2021). Amplicons were purified using Macherey-Nagel NucleoMag magnetic beads, then barcoded with a set of UNI-tagged PacBio barcodes using an asymmetric barcoding strategy (Stinson et al. 2021). Barcoded amplicons were normalized, pooled, and magnetic bead purified. Pools were sequenced at the Australian Genome Research Facility (University of Queensland, QLD, Australia) using the PacBio Sequel II system.

### Sequence processing

Sequence data was processed using mothur version 1.44.1 (Schloss et al. 2009), as previously described (Stinson et al. 2021). Subsampling was performed to 977 reads based on the size of the smallest library (not including negative controls). Taxa detected in negative controls are reported in Table S1.

### Statistical analysis

#### Bacterial culture

Each sample type was compared to baseline (T0) MOM samples using Wilcoxon signed-rank test.

#### Metataxonomic analysis

Our sequence data revealed evidence of contamination in one batch of donor milk, which was not evident via culture. These contaminating reads mapped to *Enhydrobacter* and *Stenotrophomonas*. Therefore, the experimental repeats related to this batch of donor milk (*n*=3) were excluded from metataxonomic analysis.

Alpha diversity was assessed using Shannon diversity and richness measures, generated from mothur. Differences between recolonized donor milk samples and baseline MOM samples were assessed using Wilcoxon signed-rank test.

Beta diversity was analyzed using Bray-Curtis distances, generated in mothur. Differences between recolonized donor milk samples and baseline MOM samples were assessed by performing PERMANOVA on Bray-Curtis distances using pairwise.adonis package in R, with 9999 permutations. A principal coordinate analysis (PCoA) was performed using the first two principal component axes.

Microbiome composition was assessed at the genus level due to a high level of inter-individual variability at the OTU level. Differences between recolonized donor milk samples and baseline MOM samples were assessed using Wilcoxon signed-rank test with Benjamini-Hochberg correction.

## Results

### Bacterial titers in fresh MOM vary inter-individually

**Fig. 2:**
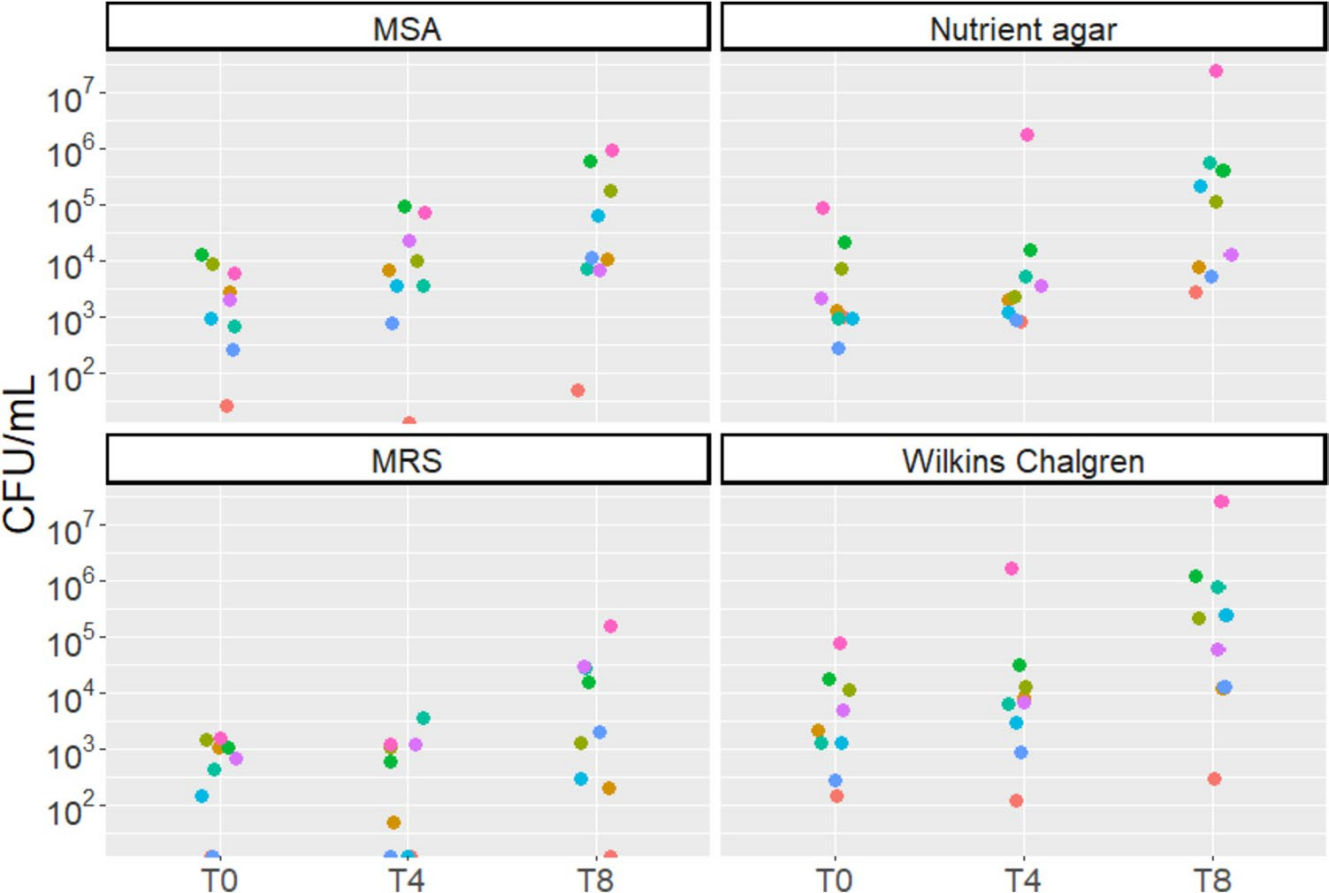
Bacterial growth (CFU/mL) of fresh mother’s own milk (MOM) samples on four culture media types over an 8-h incubation period. T0: baseline. T4: 4 h. T8: 8 h. MSA: Mannitol salt agar. MRS: acidified de Man, Rogosa, and Sharpe agar

Baseline measures of bacterial titers in freshly expressed MOM were highly variable between individuals (Fig. 2). Growth was detected on MSA, nutrient agar, and Wilkins Chalgren agar media for all baseline MOM samples (median 2000 CFU, 1250 CFU, and 2175 CFU, respectively), while growth on MRS was only detected in seven of the nine baseline MOM samples (median 700 CFU). Bacterial growth in MOM increased significantly between each time point on MSA and Wilkins Chalgren agar (MSA T0 vs T4, *P*=0.0078; MSA T4 vs T8, *P*=0.039; Wilkins Chalgren T0 vs T4, *P*=0.0078; Wilkins Chalgren T4 vs T8, *P*=0.0039). On both MRS and nutrient agar, growth significantly increased between 4 and 8 h of incubation (MRS, *P*=0.014; nutrient agar, *P*=0.0039).

### Expansion of cultivatable bacteria in DHM

**Fig. 3:**
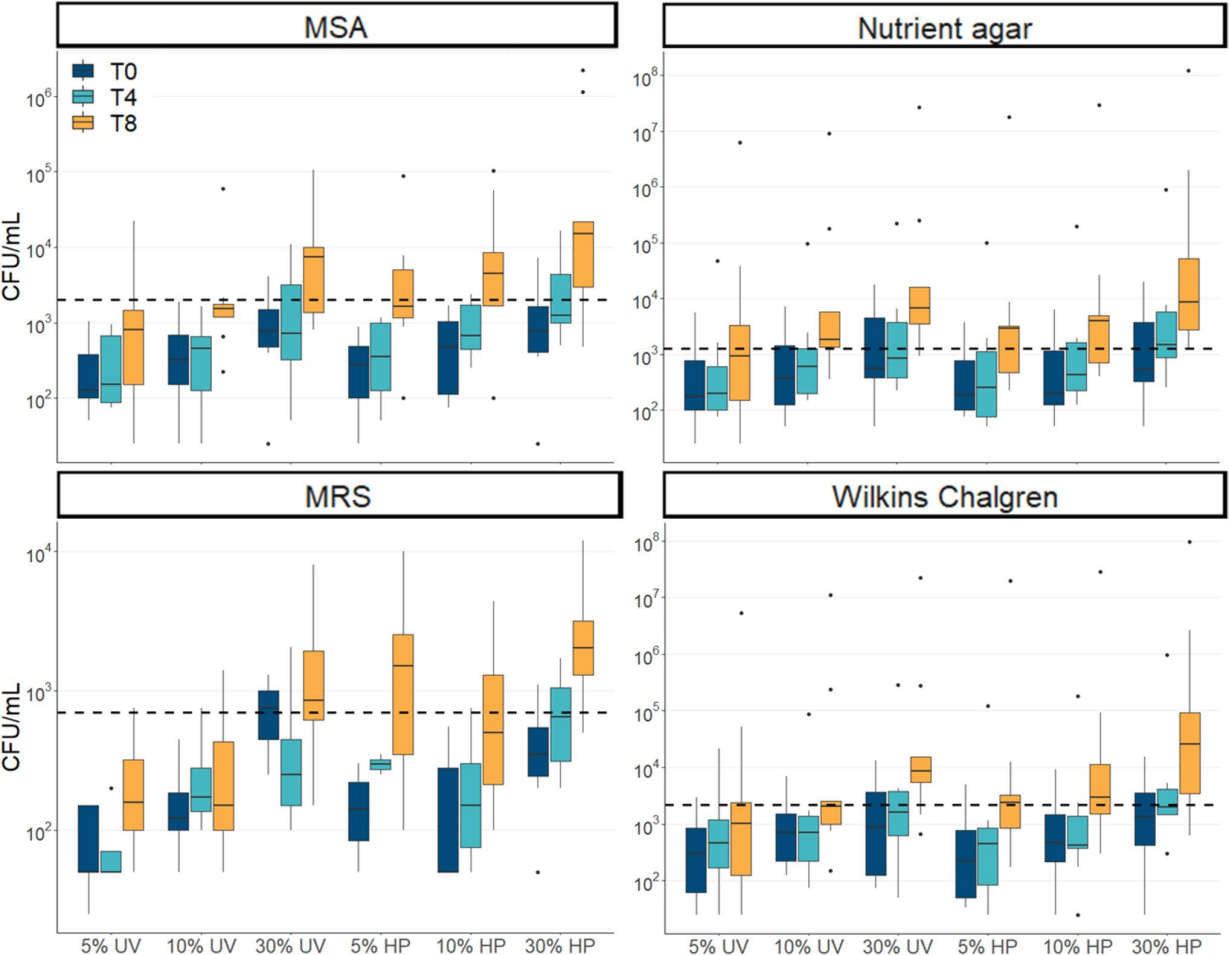
Bacterial growth (CFU/mL) of recolonized milk samples on four culture media types over an 8-h incubation period. The dashed line represents the median value for fresh (T0) MOM, the goal for recolonization. T0: baseline (dark blue boxes). T4: 4 h (light blue boxes). T8: 8 h (orange boxes). MSA: Mannitol salt agar. MRS: Acidified de Man, Rogosa, and Sharpe agar

Both UV-C-treated and Holder-pasteurized DHM samples yielded no growth across the 8-h incubation period on any of the tested culture media. In recolonized DHM samples, expansion of cultivatable bacteria was observed over time across all media tested (Fig. 3). Bacterial growth correlated well with both incubation time and percentage of MOM inoculated. Bacteria expanded more readily and rapidly in Holder-pasteurized compared to UV-C-treated donor milk, with significantly higher titers seen in Holder-pasteurized compared to UV-C-treated restored milk samples (Table S2).

Baseline MOM titers were used as the goal for recolonization. For nutrient agar, which promotes the growth of facultative aerobes such as *Streptococcus*, this was achieved after 4 h of incubation for RM-UV 10% and 30% and RM-HP 5%, 10%, and 30% and after 8 h for all samples (all *P*>0.055, Table S3). In MSA, which promotes the growth of *Staphylococcus*, restoration of baseline MOM growth was achieved after 4 h of incubation for RM-UV 30% and RM-HP 30% and after 8 h of incubation for RM-UV 5%, 10%, and 30% and RM-HP 5% and 10% (all *P*>0.19). In Wilkins Chalgren agar, which promotes the growth of anaerobic bacteria, restoration of baseline MOM growth was achieved after 4 h of incubation for RM-UV 10% and 30% and RM-HP 5%, 10%, and 30% and after 8 h of incubation for RM-UV 5%, 10%, and 30% and RM-HP 5% and 10% (all P>0.13). In MRS, which promotes the growth of lactic acid bacteria, including *Lactobacillus* and *Bifidobacteria*, restoration of MOM growth was achieved after 4 h in RM-UV 30% and RM-HP 30% and after 8 h in RM-UV 5% and 30% and RM-HP 5% and 10% (all *P*>0.052). However, after 8 h of incubation, bacterial growth in RM-HP 30% significantly overtook baseline MOM levels on MSA (*P*=0.0091), MRS (*P*=0.014), and Wilkins Chalgren agar (*P*=0.0039), suggesting that this recolonization condition results in overgrowth of bacteria. Based on these results, 4 h of incubation at 10–30% is a reasonable restoration strategy for Holder-pasteurized and UV-C-treated milk.

### MOM microbiome remains stable after inoculation into DHM

**Fig. 4:**
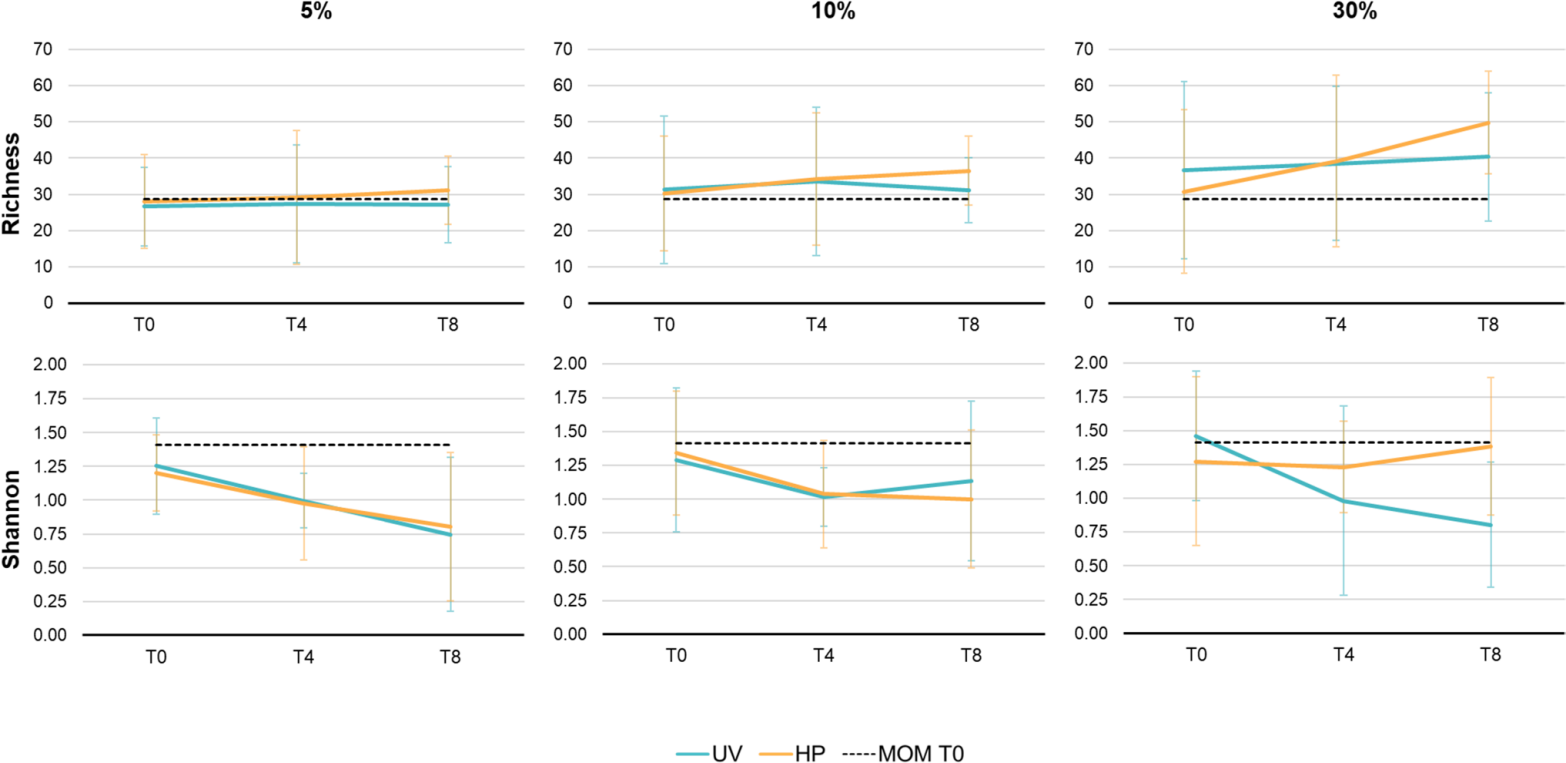
Bacterial richness and Shannon diversity in PMA-treated recolonized milk samples over time (mean±SD). Blue lines represent recolonized UV-C-treated donor milk. Orange lines represent recolonized Holder pasteurized donor milk. Dashed black lines represent the average baseline (T0) diversity of fresh MOM samples. T0: baseline. T4: 4 h. T8: 8 h

**Fig. 5:**
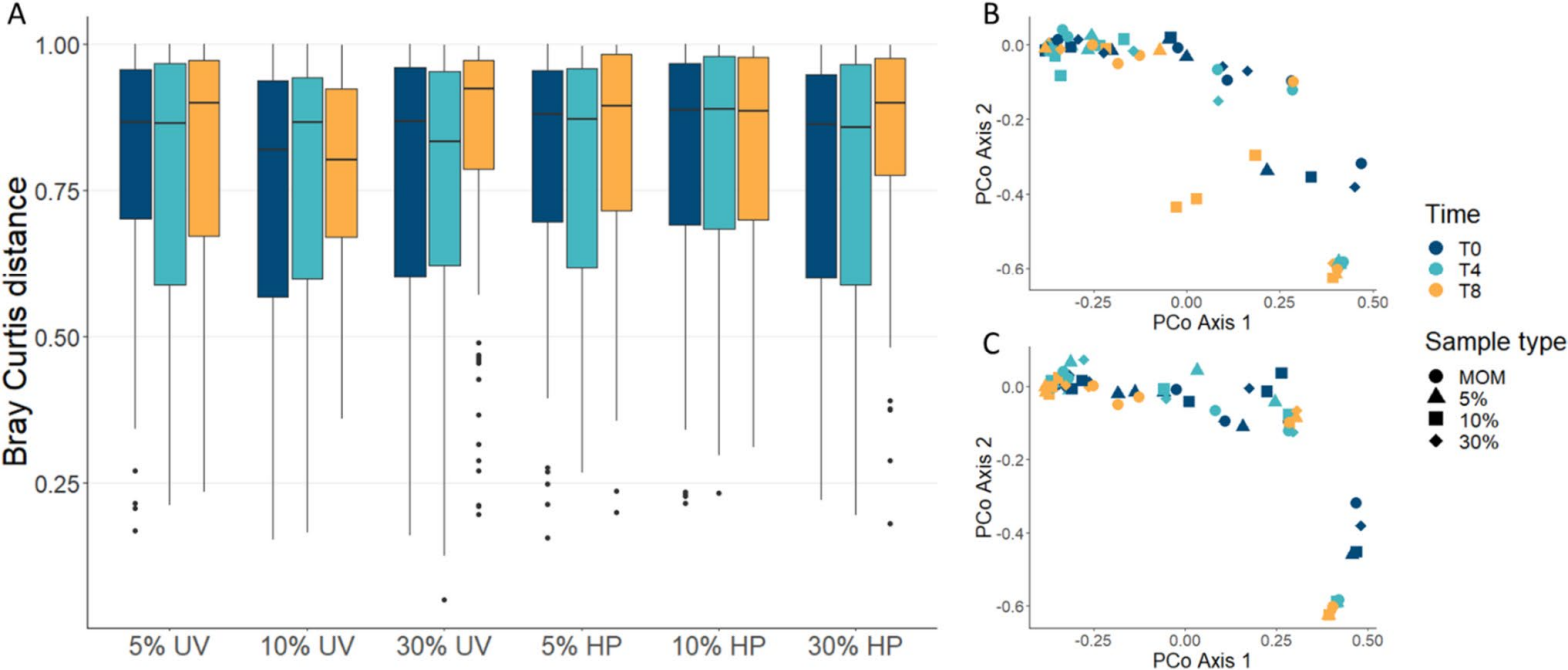
Beta diversity does not differ between baseline MOM (T0) samples and recolonized DHM samples. **A** Bray-Curtis distances from each recolonized sample type to baseline MOM samples (T0). **B** PCoA of Bray-Curtis distances between MOM and recolonized UV-treated DHM. **C** PCoA of Bray-Curtis distances between MOM and recolonized Holder-pasteurized DHM

The mean viable bacterial richness and Shannon diversity of fresh MOM samples were 28.6±10.1 and 1.4±0.5, respectively, with no significant change over time (all *P*>0.4). The viable bacterial richness and Shannon diversity of recolonized DHM samples did not differ significantly from baseline MOM levels at any time point (all *P*>0.05; Fig. 4). Further, the beta diversity of recolonized DHM samples did not differ from baseline MOM samples at any time point (PERMANOVA, all *P*>0.281; Fig. 5). At the genus level, there were no statistically significant differences between the relative abundance of each genus in baseline MOM samples and recolonized DHM samples (all *P*≥0.73; Fig. S1). Overall, the composition and diversity of recolonized DHM samples did not differ from fresh MOM samples and remained stable across the 8-h incubation period.

## Discussion

Pasteurized donor milk is void of potentially beneficial MOM bacteria. In the present study, we have demonstrated the ability to transplant MOM microbiota into pasteurized DHM. This study builds on previous such work (Cacho et al. 2017; Mallardi et al. 2021; Torrez Lamberti et al. 2021) by comparing recolonization success in DHM treated by two different pasteurization methods: UV-C treatment and Holder pasteurization. Holder pasteurization was examined as it is clinically relevant, being widely used in milk banks globally. UV-C treatment is a more recent development in the field (Christen et al. 2013a; Christen et al. 2013b) that destroys bacteria and viruses in DHM (Christen et al. 2013a; Christen et al. 2013b; Lloyd et al. 2016; Stinson et al. 2023) while conserving the biochemical composition of milk (Christen et al. 2013a; Christen et al. 2013b), which is diminished by Holder pasteurization. Our culture results demonstrate that bacteria grew more rapidly in Holder-pasteurized compared to UV-C-treated DHM. These results were contrary to the expectation that MOM bacteria would better expand in UV-C-treated DHM due to the compositional similarity with MOM. However, these findings align with the observed loss of bioactive antimicrobial factors in Holder-pasteurized milk (Peila et al. 2016). UV-C treatment has been shown to retain the important antimicrobial proteins lactoferrin, lysozyme, and secretory immunoglobulin A (sIgA), with retention levels of 87%, 75%, and 89%, respectively, compared to Holder pasteurization which retains merely 9%, 41%, and 49%, respectively (Christen et al. 2013a). Therefore, the higher degree of antimicrobial action in UV-C-treated DHM may have suppressed bacterial growth in these samples to a greater degree than Holder-pasteurized milk. Importantly, the role of human milk is not simply to supply bacteria to the infant gut; rather, maternal bacteria are delivered alongside prebiotics (human milk oligosaccharides) and a range of antimicrobial proteins (lysozyme, lactoferrin, sIgA), which likely function to modulate early microbial colonization. Indeed, recent data indicate that approximately 40% of bacteria in human milk are IgA coated (Dzidic et al. 2020), suggesting an important role for milk immunological proteins. Thus, adding MOM bacteria into Holder-pasteurized DHM, which is devoid of these important immunological proteins, may confer a risk to vulnerable infants. Delivery of MOM bacteria alongside endogenous immune- and microbial-modifying bioactives may be a better strategy for establishing the infant gut microbiome.

Based on our culture results, restoration of DHM with 10–30% MOM, incubated for 4 h, is a reasonable strategy. Importantly, bacterial growth in high inoculum samples (30% MOM) far surpassed baseline MOM levels after 8 h of incubation. This kind of florid growth may be undesirable in a NICU setting, where conservative recolonization strategies with lower bacterial loads may be desirable. Our culture results align well with those of previous studies in which Holder-pasteurized DHM has been recolonized with fresh (Cacho et al. 2017; Mallardi et al. 2021) or frozen (Torrez Lamberti et al. 2021) milk. These studies have reported that 10–30% recolonized milk incubated for 2–4 h yielded similar bacterial levels to MOM, with prolonged incubation in high inoculum samples resulting in bacterial loads that were higher than those of baseline MOM samples.

Our metataxonomic results demonstrated the stability of the MOM bacterial profile when transplanted into pasteurized DHM, with no significant differences in composition between baseline MOM samples and recolonized DHM samples. Importantly, our study is the first to use viability-coupled sequencing to assess the microbiome of recolonized DHM. Previous metataxonomic studies of recolonized DHM have been confounded by background DNA from non-viable organisms in pasteurized DHM. Indeed, in such studies, the alpha diversity of pasteurized DHM samples was no different to that of fresh MOM (Cacho et al. 2017; Torrez Lamberti et al. 2021), and the metataxonomic profile of recolonized DHM very much reflected that of pasteurized DHM (Mallardi et al. 2021). By eliminating DNA from non-viable organisms found in pasteurized DHM, we have been able to assess recolonization efficacy with a far greater level of sensitivity. However, even non-viable organisms may have an impact on infant health, with the recent recognition of para-probiotics, or “ghost probiotics” (Monteiro et al. 2023; Siciliano et al. 2021). Para-probiotics are non-viable (often heat-inactivated) microbial cells or microbial components which confer a health benefit when consumed. Host recognition of non-viable bacterial components or products can illicit inflammatory or immune responses. As such, many studies have demonstrated equal efficacy of live probiotics and heat-inactivated para-probiotics (Monteiro et al. 2023; Siciliano et al. 2021), with experts calling for these to be trialed in preterm infants, a population in which non-viable probiotics may be safer than traditional probiotics (Deshpande et al. 2018). Indeed, fresh human milk is rich in non-viable bacteria, which may be important for infant immune programming (Stinson et al. 2021). As such, the “ghost microbiota” harbored by pasteurized DHM may have immunological impacts in the preterm infant.

The alpha diversity of recolonized DHM samples did not differ from baseline MOM samples at any time point. This result was anticipated, as each aliquot of MOM that was inoculated into each DHM sample would be expected to subsample a similar level of diversity. Further, sample richness would not be expected to increase over time, as new taxa are not being created. A decrease in taxonomic richness may have been observed if taxa were dying over time; however, this was not apparent. Therefore, while our culture-based results indicated an increase in total bacterial quantity over time, our sequence data showed that diversity remained stable.

Compared to specific probiotic supplementation with beneficial infant gut taxa such as *Bifidobacterium* spp. (commonly used in the NICU for preterm infants (Athalye-Jape et al. 2022; Beck et al. 2022; Beghetti et al. 2021)), transplantation of the global MOM microbiome is likely to have different biological effects. On one hand, targeted probiotic supplementation ensures delivery of proven beneficial microbes while avoiding the possibility of exposure to potential pathogens harbored in the maternal microbiome. Conversely, exposure to a broad range of maternal microbes may be beneficial for early immune programming, and more closely resemble exposures experienced by exclusively MOM-fed infants. Regardless, safety is likely to be a central concern when considering administration of recolonized DHM to vulnerable infants, particularly in light of data from this and previous studies showing rapid expansion of bacteria in pasteurized DHM (Cacho et al. 2017; Mallardi et al. 2021; Torrez Lamberti et al. 2021). Thus, microbiological monitoring may be required if *in vitro* studies such as our own are ever to be translated clinically.

There are a number of important considerations for translation of this method, such as ensuring the safety of recolonized DHM for consumption by infants and weighing the potential benefits against the time and expense of the method. As already discussed, ensuring the safety of recolonized DHM likely involves limiting incubation time to prevent overgrowth of bacteria. Of note, one MOM sample in our study had exceptionally high levels of growth on Wilkins Chalgren and nutrient agar (Fig. 2), with similar levels of growth observed in UV-C-treated DHM inoculated with this sample, and even higher levels of growth seen in Holder-pasteurized DHM inoculated with this sample (Fig. 3). This raises the question of whether undesirable bacteria in MOM could inadvertently expand in pasteurized DHM using this technique. Microbiological screening for potential pathogens and monitoring of total bacterial load would likely be required if this method is to be implemented clinically. Recolonization of DHM may also result in transfer of antimicrobial resistance genes to the infant, though this may also occur through MOM feeding (Samarra et al. 2023). Another consideration is the volume of MOM required for recolonization. In our study and others, 10–30% inoculums were able to restore bacterial loads seen in fresh MOM after 4 h of incubation. For a 1500-g infant with a 24-h milk requirement of 190 mL, 19–57 mL of MOM would be sufficient to prepare a day’s worth of recolonized DHM. If the mother is able to produce 100 mL per day, as little as 9 mL may be required to recolonize the remaining 90 mL of DHM. In such a scenario, it would be important to weigh the cost and benefit of the time and expense required to recolonize 90 mL of DHM per day.

In this small-scale *in vitro* study, we have demonstrated the feasibility of transplanting MOM microbiota into both UV-C-treated and Holder-pasteurized DHM. While both methods were able to recapitulate the MOM bacterial profile, bacterial load increased more rapidly in Holder-pasteurized DHM, likely due to the lack of antimicrobial proteins. Our findings add to previous work in the field (Cacho et al. 2017; Mallardi et al. 2021; Torrez Lamberti et al. 2021), highlighting the potential to grow bacteria from small volumes of MOM in pasteurized DHM, and expand on this work by testing a novel pasteurization method, and utilizing viability-coupled sequencing. While our results are promising, we caution that further safety testing is necessary to refine the method and ensure bacterial loads are suitable for administration to vulnerable infants.

## Supplementary Information

ESM 1

## Data Availability

Raw FASTQ sequence files and metadata are available at NCBI SRA (BioProject ID: PRJNA1013614).
