# Supplementary material for: Milk microbiome transplantation: recolonizing donor milk with mother's own milk microbiota
Source: Appl Microbiol Biotechnol. 2024 Jan 9;108:74. doi: 10.1007/s00253-023-12965-8 (PMC10776751; doi:10.1007/s00253-023-12965-8)
Supplement: Supplementary file 1 [file 253_2023_12965_MOESM1_ESM.pdf]

Supplementary data

Journal: Applied Microbiology and Biotechnology

Milk Microbiome Transplantation: Recolonizing Donor Milk with Mother's Own Milk Microbiota

Lisa F. Stinson[1*], Jie Ma[1], Ching Tat Lai[1], Alethea Rea[2], Sharon L. Perrella[1], and Donna T. Geddes[1]

[1] School of Molecular Sciences, The University of Western Australia, Perth, Australia
[2] Mathematics and Statistics, Murdoch University, Perth, Australia

* Corresponding author
lisa.stinson@uwa.edu.au
+6148 6488 3200

Supplementary data

Supplementary Table 1: Genera detected in negative extraction controls (EC1-9) and negative PCR (no template) controls (NTC1-5). Values are read numbers.

Genus	EC1	EC2	EC3	EC4	EC5	EC6	EC7	EC8	EC9	NTC1	NTC2	NTC3	NTC4	NTC5
Acinetobacter	1	8	8	0	2	2	0	9	4	5	2	2	1	1
Azomonas	0	0	0	0	1	0	0	0	0	0	1	0	0	0
Bacillus	0	14	1	0	0	0	0	0	0	2	1	0	0	0
Blastococcus	0	0	0	0	2156	0	0	0	0	0	0	0	0	0
Bradyrhizobium	0	1	1	0	0	0	0	0	0	1	0	2	0	0
Caldimonas	0	0	1	0	0	0	0	0	0	0	0	0	0	0
Chryseobacterium	0	0	4	0	1	1	0	0	0	6	2	0	0	0
Corynebacterium	0	0	3	1	2	1	0	0	0	3	0	0	0	0
Curvibacter	0	0	2763	1	0	476	0	0	0	0	0	0	0	0
Cutibacterium	0	0	4	0	0	0	0	0	2	3	0	1	6	0
Delftia	0	0	0	0	0	0	101	0	0	0	0	0	0	0
Enhydrobacter	0	8	54	2855	17	20	0	0	0	55	19	21	0	0
Enterobacter	1	0	3	0	2	8	0	30	0	461	0	0	14	1
Gemella	0	2	2	0	0	1	0	1	0	8	0	0	0	0
Granulicoccus	0	0	0	0	0	0	0	0	0	1	0	0	0	0
Ideonella	0	0	4600	0	0	0	0	0	0	0	0	0	0	0
Lactobacillus	0	0	0	0	0	0	0	0	0	2	0	1	0	0
Lawsonella	0	0	0	33	0	0	0	0	0	0	0	0	0	0
Methylarcula	0	0	0	0	0	0	0	0	0	0	0	1	0	0
Methylobacterium-Methylorubrum	0	0	0	0	0	20	0	0	0	0	0	0	0	0
Pantoea	4	0	0	0	2	3	0	0	1	0	0	0	0	0
Planifilum	0	1	0	0	0	0	0	0	0	0	0	0	0	0
Propioniferax	0	0	0	0	0	0	0	0	0	4	0	0	0	0
Pseudarthrobacter	0	0	0	1	0	0	0	0	0	0	0	0	0	0

Pseudomonas	3	0	17	0	8	7	0	2	5	24	7	3	4	0
Ralstonia	0	37	1	0	0	0	0	0	0	0	0	0	0	0
Serratia	0	0	1	0	0	0	0	0	0	0	0	0	0	0
Skermanella	0	0	0	0	0	0	0	0	0	1	0	0	0	0
SN8	0	1	0	2	1	0	0	0	0	0	0	0	1	0
Sphingomonas	0	1	0	0	0	0	0	0	0	0	0	0	0	0
Staphylococcus	0	302	86	92	17	17	0	32	41	110	25	42	39	3
Stenotrophomonas	0	124	14	2	6	5	0	1	0	17	15	22	0	0
Streptococcus	0	380	19	3	1	1	0	1	1	62	16	0	0	0
Thermoactinomyces	0	0	0	213	0	0	0	0	0	0	0	0	0	0
Unclassified *Bacillaceae*	0	2	0	1	2	0	0	1	1	6	1	1	1	0
Unclassified *Comamonadaceae*	0	6194	17	0	0	0	0	0	0	0	0	2	0	0
Unclassified *Enterobacteriaceae*	1	0	3	0	2	2	0	7	3	13	1	0	3	0
Unclassified *Erwiniaceae*	0	0	0	0	0	1	0	0	0	0	0	0	0	0
Unclassified *Micrococcaceae*	0	0	0	1	0	0	0	0	0	0	0	0	0	0
Unclassified *Moraxellaceae*	0	0	1	0	0	1	0	0	1	0	0	1	1	0
Unclassified *Propionibacteriaceae*	0	0	0	0	0	0	0	0	0	1107	0	0	0	0
Unclassified *Pseudomonadaceae*	0	0	1	1	1	2	0	0	0	0	3	0	1	0
Unclassified *Rhizobiaceae*	0	0	0	0	0	0	0	0	0	1	0	0	0	0
Unclassified *Staphylococcaceae*	0	0	0	0	0	0	0	0	0	1	0	1	0	0
Unclassified *Streptococcaceae*	0	0	0	0	0	1	0	0	0	0	0	0	0	0
Unclassified *Weeksellaceae*	0	0	0	1	0	1	0	0	0	1	0	0	0	0
Unclassified *Xanthobacteraceae*	0	0	0	0	0	0	0	0	0	0	0	0	1	0
Unclassified *Xanthomonadaceae*	0	0	0	0	0	0	0	0	0	0	1	0	0	0
Yokenella	0	0	0	0	0	0	0	0	0	2	0	0	0	0

Supplementary Table 2: Comparison of bacterial titers in recolonized Holder pasteurized and recolonized UV-C treated milk samples. Data are P values derived from Wilcoxon signed-rank test. * Denotes significant P values.

% Inoculation	Time	MSA	Nutrient agar	MRS	Wilkins Chalgren
5%	T0	0.44	0.085	0.20	0.27
	T4	0.025 *	0.12	0.58	0.94
	T8	0.16	0.30	0.11	0.098
10%	T0	0.94	0.61	0.89	0.028 *
	T4	0.013 *	0.83	0.58	0.14
	T8	0.074	0.57	0.17	0.20
30%	T0	1	0.83	0.60	0.050
	T4	0.098	0.41	0.29	0.13
	T8	0.055	0.21	0.036 *	0.027 *

Supplementary Table 3: Comparison of bacterial titers in fresh (T0) MOM and recolonized milk samples. Data are P values derived from Wilcoxon signed-rank test. * Denotes significant P values.

Pasteurization method	% Inoculation	Time	MSA	Nutrient agar	MRS	Wilkins Chalgren
UV	5%	T0	0.0039 *	0.0039 *	0.023 *	0.0039 *
		T4	0.0039 *	0.0091 *	0.023 *	0.0039 *
		T8	0.36	1	0.052	0.82
	10%	T0	0.014 *	0.0039 *	0.022 *	0.0039 *
		T4	0.014 *	0.16	0.022 *	0.13
		T8	0.57	0.73	0.023 *	1
	30%	T0	0.0039 *	0.0039 *	0.076	0.0039 *
		T4	0.19	0.30	0.074	0.34
		T8	0.20	0.20	0.17	0.25
HP	5%	T0	0.0039 *	0.0091 *	0.022 *	0.0039 *
		T4	0.0039 *	0.098	0.022 *	0.13
		T8	0.91	0.91	0.93	1
	10%	T0	0.0039 *	0.0039 *	0.022 *	0.0039 *
		T4	0.014 *	0.13	0.023 *	0.13
		T8	0.30	0.65	0.93	0.65
	30%	T0	0.0039 *	0.0039 *	0.023 *	0.0090 *
		T4	0.73	0.48	0.21	0.77
		T8	0.0091 *	0.055	0.014 *	0.0039 *

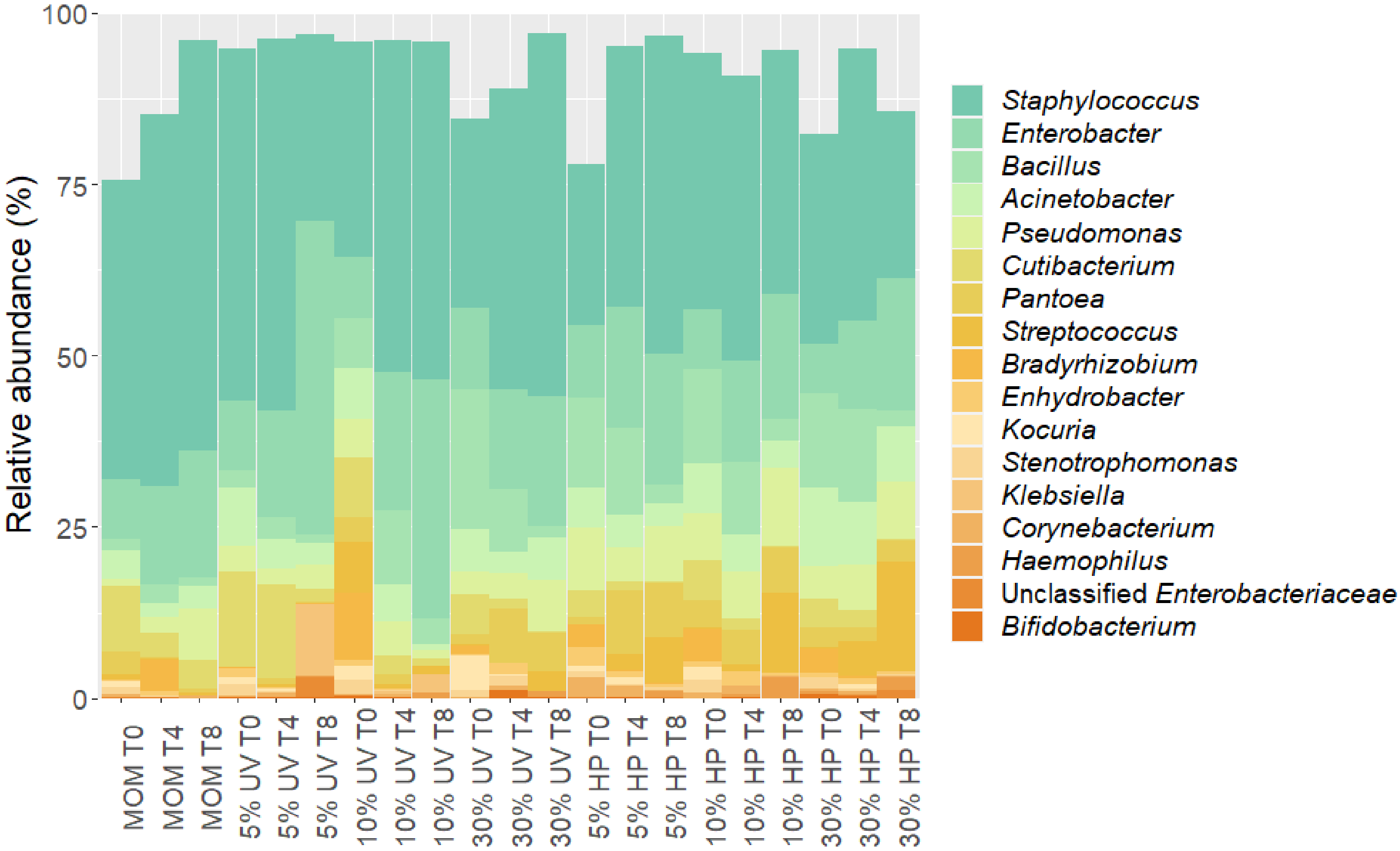

Supplementary Figure 1: Mean relative abundance the major viable genera (>0.05% relative abundance) across each sample type. MOM: mother's own milk. T0: baseline. T4: 4 hours. T8: 8 hours.
